# Effect of β-Glucan and Black Tea in a Functional Bread on Short Chain Fatty Acid Production by the Gut Microbiota in a Gut Digestion/Fermentation Model ^†^

**DOI:** 10.3390/ijerph16020227

**Published:** 2019-01-15

**Authors:** Abbe M. Mhd Jalil, Emilie Combet, Christine A. Edwards, Ada L. Garcia

**Affiliations:** 1 Faculty of Health Sciences, School of Nutrition and Dietetics, Kampus Gong Badak, Universiti Sultan Zainal Abidin, Kuala Terengganu 21300, Terengganu Darul Iman, Malaysia; abbemaleyki@unisza.edu.my; 2 Human Nutrition, School of Medicine, College of Medical, Veterinary and Life Sciences, University of Glasgow, New Lister Building, Glasgow Royal Infirmary, 10-16 Alexandra Parade, Glasgow G31 2ER, UK; Emilie.CombetAspray@glasgow.ac.uk (E.C.); Christine.Edwards@glasgow.ac.uk (C.A.E.)

**Keywords:** gut microbiota, beta glucan, black tea, phenolic acids, short chain fatty acids, acetate, propionate, butyrate, in vitro fermentation, in vitro digestion

## Abstract

β-Glucan and black tea are fermented by the colonic microbiota producing short chain fatty acids (SCFA) and phenolic acids (PA). We hypothesized that the addition of β-glucan, a dietary fiber, and tea polyphenols to a food matrix like bread will also affect starch digestion in the upper gut and thus further influence colonic fermentation and SCFA production. This study investigated SCFA and PA production from locally developed breads: white bread (WB), black tea bread (BT), β-glucan bread (βG), β-glucan plus black tea bread (βGBT). Each bread was incubated in an in vitro system mimicking human digestion and colonic fermentation. Digestion with α-amylase significantly (*p* = 0.0001) increased total polyphenol and polyphenolic metabolites from BT bread compared with WB, βG, and βGBT. Total polyphenols in βGBT remained higher (*p* = 0.016; 1.3-fold) after digestion with pepsin and pancreatin compared with WB. Fermentations containing βG and βGBT produced similar propionate concentrations ranging from 17.5 to 18.6 mmol/L and total SCFA from 46.0 to 48.9 mmol/L compared with control WB (14.0 and 37.4 mmol/L, respectively). This study suggests that combination of black tea with β-glucan in this functional bread did not impact on SCFA production. A higher dose of black tea and β-glucan or in combination with other fibers may be needed to increase SCFA production.

## 1. Introduction

The gut microbiota is sustained by non-digested or non-absorbed food components. Two of the key food constituents metabolized by the colonic bacteria are dietary fiber and plant polyphenols. Some starch also escapes digestion and is available for fermentation in the colon. Starch digestion could be influenced by the food matrix including dietary fiber. It is important to understand the impact of combining food ingredients in functional foods on the supply of nutrients to the colonic microbiota and the subsequent production of bioactive molecules including short chain fatty acids (SCFA) and phenolic acids (PA).

Starch and gluten are key components in bread that undergo physicochemical changes during the bread making process [1]. Bread is commonly consumed in Western countries and can be manipulated for further development of functional properties [2]. The addition of soluble fiber such as guar gum (galactomannan) to bread reduced in vitro starch hydrolysis (approximately 31% against control wheat bread) by preventing starch-α-amylase interactions [3]. Our previous findings demonstrated that incorporating β-glucan into bread could help ‘preserve’ the starch granules compared with normal wheat bread [4]. The presence of β-glucan conserved the starch structure and/or reduced the surface area for α-amylase-starch interaction. These contributed to reduced starch hydrolysis which could increase delivery of substrate to the colon for the gut microbiota.

β-Glucan is a soluble dietary fiber with mixed β-(1→3) and β-(1→4) glucosidic linkages. Four gram of β-glucan per 30 g available carbohydrate has been approved by the European Food Safety Authority (EFSA) to reduce the glycemic response without disproportionally increasing the postprandial insulinemic response [5]. Studies have shown that puddings and snack bars prepared with β-glucan positively reduced post-prandial glycemic (17% versus control) and insulin concentrations (27% versus control) [6,7]. Mechanistically, low and high molecular weight β-glucan are fermented by the intestinal microbiota, resulting in production of the short chain fatty acids (SCFA) acetate, propionate, and butyrate [8]. Propionate acts as a precursor in hepatic glucose production and is involved in appetite regulation through the stimulation of hepatic nerves [9]. The consumption of inulin propionate ester (10 g/day) in healthy humans increased fullness by 14%, when compared with an inulin control [10]. However, the dose (10 g/day) used in this study was very high and is not attainable from normal dietary intake. The question remains on how to increase the level of propionate in our body or whether the presence of another bioactive component such as polyphenols can synergistically increase the health benefit of β-glucan.

Polyphenols found in foods such as fruits, vegetables, tea, and coffee are important bioactive compounds associated with beneficial health effects such as reduction in blood pressure [11], cardiovascular risk factors [12], and type 2 diabetes [13]. Polyphenols in fruits, vegetables, or cereals are usually located in the plant cell walls or bound within the food matrix which reduces their bioavailability [14]. However, polyphenols in beverages such as black tea are more ‘readily’ available and can be used in formulations together with β-glucan. Black tea is an ideal choice for testing synergistic effects of fiber and polyphenols because of its wide consumption. The average black tea consumption in Europe is four cups/day (1000 mL) in males and three cups/day (865 mL) in females [15]. Acute black tea intake (250 mL) containing 350 mg total polyphenols reduced postprandial glycaemia by 52% in a randomized-crossover human intervention [8]. An in vivo animal study suggested that black tea (5 mg/kg body weight) reduced glucose levels when compared with a drug (acarbose) commonly used for type 2 diabetes mellitus treatment (T2DM) [16]. Mechanistically, the presence of bioactive polyphenols inhibited α-amylase and/or α-glucosidase enzymic activity and therefore the glycemic response blunts [17].

The health benefits of black tea are due to the presence of small and large molecular weight polyphenols [9]. Black tea is rich in quercetin (10–25 mg/L), kaempferol (7–17 mg/L), and myricetin (2–5 mg/L) and high molecular weights theaflavins (TF) and thearubigins (TR) [18,19]. TF and TR are responsible for the pigments of black tea, derived by enzymatic oxidation during tea fermentation [19]. These large complexes are poorly digested and most tea polyphenols escape to the colon where they are metabolized by the gut bacteria. In humans, tea flavan-3-ols reached the colon (50–70% of ingested dose) after drinking a cup (300 to 500 mL) of green tea [20,21,22]. The polyphenols may be further metabolized into SCFA by the gut microbiota. Unno and Osakabe demonstrated that 10 g/kg black tea extract increased caecal propionate production in rats compared with green tea and the control [19].

Food components (e.g., fiber, polyphenols) can interact in several ways when eaten as a separate meal, but if combined in the same food matrix in foodstuffs such as bread that are heavily transformed during food processing, these interactions may be more complex. Tea polyphenols can interrupt amylose recrystallisation that results in a more rigid structure more resistant to enzymatic action [23]. Soluble β-glucan addition to a polyphenol containing food matrix has the potential to ‘trap’ high molecular weight black tea polyphenols within the starch granules to form a complex and substrate for colonic bacteria fermentation. We aimed to investigate the effects of combining black tea and β-glucan in a food matrix on polyphenol release and SCFA production in an in vitro model mimicking human digestion and fermentation.

## 2. Materials and Methods

### 2.1. Study Design

**Figure 1:**
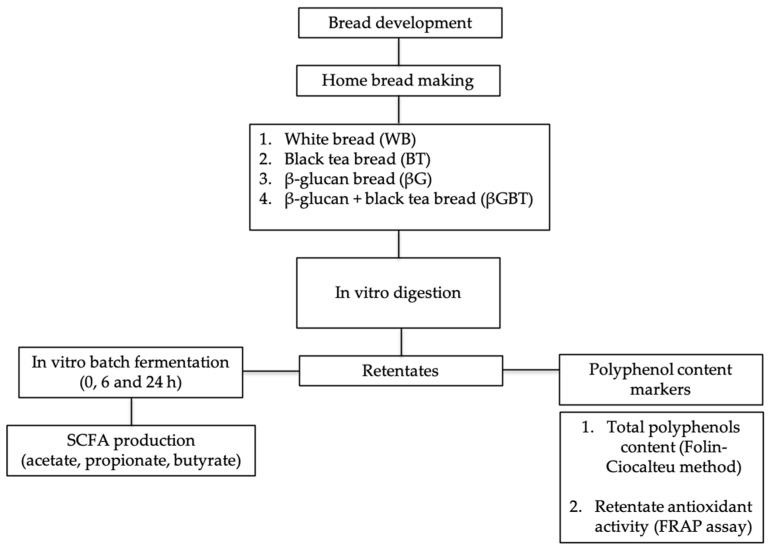
Study diagram for development of bread, in vitro digestion, and fermentation. SCFA, short chain fatty acids; FRAP, ferric-reducing availability of plasma.

This was an in vitro study using two experimental approaches: (1) digestion of breads using a model mimicking human gastric and duodenal phases of digestion to measure antioxidant potential and (2) batch culture fermentations of predigested breads using fecal samples collected from healthy donors (Figure 1).

### 2.2. Bread Development

#### 2.2.1. Materials

White wheat flour, yeast for baking, unsalted butter, and dried skimmed milk were purchased from WM Morrisons Supermarkets PLC (Bradford, UK). β-Glucan concentrate (from barley) (Glucagel^TM^) was purchased form DKSH (Quai du Rhône, France) and freeze dried black tea from Tata Global Beverages GB LTD (Greenford, UK). The black tea contained 452.2 (18.9) mg gallic acid equivalents/100 mL.

#### 2.2.2. Bread Preparation

Breads were prepared using our previous recipe [4]. One portion of black tea (BT) bread (111 g) and β-glucan plus black tea (βGBT) bread (153 g) provided 30% of polyphenols that could be obtained from a cup (250 Ml) of black tea [24,25]. All ingredients (Table 1) were weighed and transferred into a baking pan. Bread was baked using a domestic bread maker (Morphy Richards Ltd., South Yorkshire, UK) set as follows: kneading (10 min), rising (20 min), kneading (15 min), rising (40 min), and baking (65 min), for a total baking time of 2 h 30 min. More water was added to β-glucan breads to compensate for additional water uptake by β-glucan [26].

### 2.3. In Vitro Digestion Model

The in vitro digestion models mimicked the human gastro intestinal (GI) tract as described in detail by Aura et al. [27]. The aim of this procedure was to remove digestible starch, protein, and fat. Following digestion, retentates were freeze-dried and used for antioxidant potential measurements and as substrates for in vitro batch fermentation. Briefly the procedure was as follows:

(i) *Oral phase*: Bread samples containing 50 mg available carbohydrate were incubated with α-amylase (50 U/sample) (Sigma-Aldrich, Dorset, UK) at 37 °C for 5 min (mimicking oral chewing).
(ii) *Gastric phase*: The pH of the mixture was adjusted to 2.5 using HCL (0.15 M, pH 2.5) and incubated with pepsin (0.7 mL, 2 mg/mL in 0.02 M HCl) at 37 °C for 2 h in a shaking water bath.
(iii) *Small intestine (duodenum and ileum) phase*: The pH was adjusted to 7.0 using sodium hydroxide (6 M). A porcine extract bile acid (Sigma B8631) (2.7 mL, 150 ng/mL in 0.15 M sodium bicarbonate) and pancreatin (mixture of amylase, proteases and lipase, 2.7 mL, 75 mg/mL in 0.15 M sodium bicarbonate) (Sigma-Aldrich, Dorset, UK) were added and incubated at 37 °C for 4 h on a shaking water bath. Absorption in the small intestine was simulated after digestion using dialysis tubing (molecular weight 500–1000 Dalton cut-off, 35 cm long with flat width of 31 mm and diameter of 20 mm) (Spectrum Laboratories, Rancho Dominguez, CA, USA) for 6 h in 2 L distilled water.
(iv) The non-digested/dialyzed products (retentate) were carefully removed from the dialysis tube and freeze-dried. The freeze-dried sample was used as substrate in the in vitro fermentation model.

### 2.4. Determination of Total Polyphenols

Total polyphenol (crude) released from breads after the in vitro digestion was estimated using the method described by Slinkard and Singleton [28]. In short, freeze-dried dialyzed samples (20 μL) were mixed with distilled water and diluted using Folin-Ciocalteu reagent. The mixture was left for 5 min at room temperature and 70 µL of 6% (*w*/*v*) Na_2_CO_3_ was added and left for a further 90 min at room temperature before reading the absorbance at 765 nm with a spectrophotometer (Multiskan^®^ Spectrum, Thermo Labsystems, Vantaa, Finland). Gallic acid in the range of 50–1000 μg/mL was used as the standard. Gallic acid equivalents (GAE, µg/g retentate) were used to report total polyphenol content.

### 2.5. Antioxidant Potential in Retentates—Ferric Reducing Ability of Plasma (FRAP) Assay

The assay was conducted on the freeze-dried dialyzed samples from the in vitro digestion as described by Benzie and Strain [29]. The FRAP reagent was prepared by mixing 10 mM 2, 4, 6-Tri(2-pyridyl)-s-triazine (TPTZ) in 40 mM HCl, 20 mM FeCl_3_ and 0.3 M acetate buffer (pH 3.6) in a 1:1:10 ratio, this was warmed and read for absorbance at time 0 using a spectrophotometer (Multiskan^®^ Spectrum, Thermo Labsystems, Vantaa, Finland). Samples (100 µL) were added to 1.8 mL of FRAP reagent and 100 µL of deionized water followed by a 4 min incubation at 37 °C. The final absorbance was read at 593 nm and subtracted from the initial absorbance to report total FRAP value (μg Fe^2+^ equivalents/g retentate).

### 2.6. In Vitro Batch Fermentation

In vitro batch fermentation mimic colonic activity and was performed using a standardized method described by Jaganath et al. [30]. This measured the fermentability of starch and the polyphenols-β-glucan complex from breads after digestion. Fecal samples obtained from four healthy Caucasian individuals (mean age, 26; SD 4 years) who had not taken any antibiotics in the past six months and had no gastrointestinal problem. The fecal samples were used for batch fermentation within 2 h of passage. The College of Medical, Veterinary and Life Sciences Ethics Committee, University of Glasgow approved the study protocol (Application No.: 2011023). Signed informed consent was obtained from all individuals prior to sampling.

In summary, the in vitro batch fermentation used a medium of tryptone, macro and microminerals in a buffer with a redox indicator at pH 7.0. [30]. All glassware and sampling apparatus were sterilized before use. The fermentation medium was boiled to degas the solution and then cooled to 37 °C in oxygen free nitrogen (OFN). This was mixed with fecal slurry prepared from fresh feces homogenized in phosphate buffer 32% (*w*/*v*). The slurry (5 mL) was mixed with fermentation medium (42 mL) and reducing solution (2 mL) in a sterilized McCartney bottle. The bottles were sealed and again purged with OFN for 1 min and placed in a shaking water bath at 37°C to mimic conditions in the colon. Aliquots of fermentation solution (3 mL) were taken from the fermentation vessels through self-sealing lids with sterile syringes and needles at 0, 6, and 24 h for the measurement of pH and SCFA (acetate, propionate, and butyrate). The pH was not strictly controlled throughout the procedure but allowed to drop in a similar manner to the pH in the proximal colon where low pH is compensated by absorption of SCFA and secretion of bicarbonate. The pH range we recorded has been detected in the proximal colon during active fermentation in humans and in pigs [31,32].

SCFA were estimated by an established method [33]. A mixture of internal standard (100 μL; 3-methyl-n-valeric acid) and 100 μL orthophosphoric acid was added to the fermentation fluid (800 μL) and. The mixture was mixed rigorously followed by extraction with 3 mL of diethyl ether. The upper phase was collected and pooled in 15 mL tubes. The extractions were then analyzed by gas chromatography with a flame ionization detector (GC-FID) (Thermo Quest Ltd., Manchester, UK) equipped with a Zebron ZB-Wax capillary column (15 m × 0.53 mm id) (Phenomenex, Cheshire, UK). Data were analyzed by Chrom-Card software (Thermo Quest, Milan, Italy). Individual SCFA were identified by comparing retention times with authentic standards (acetic acid, propionic acid, isobutyric acid, n-butyric acid, isovaleric acid, n-valeric acid, n-hexanoic acid, heptanoic acid, and n-octanoic acid).

### 2.7. Statistical Analysis

SPSS software (SPSS version 22.0, SPSS Inc., Chicago, IL, USA) was used for data analysis. Normality was determined using Shapiro Wilk test. Data are expressed as mean ± standard deviation, SD). One-way ANOVA with Bonferroni correction assessed the mean differences between groups (log transformed data). Repeated measures ANOVA tested differences between 0, 6, and 24 h. Statistical significance was accepted at *p* < 0.05.

## 3. Results

### 3.1. Total Polyphenols and Antioxidant Potential

The release of polyphenols (crude) following in vitro digestion is shown in Table 2. Total polyphenol content of black tea bread (BT) was higher (1.6-fold, *p* = 0.0001) with 361.1 ± 20.0 μg GAE/g retentate compared with white bread (WB) (222.6 ± 63.0 μg GAE/g retentate). The release of polyphenols after digestion with α-amylase at 0 h ranged from 222.6 ± 63.0 to 361.1 ± 40.0 μg GAE/g retentate. Following gastric digestions with pepsin and pancreatin and 6 h dialysis in retentates resulted in an increase in polyphenol content of βGBT (1.3-fold) (*p* = 0.016) compared with WB.

The results of the FRAP assay in retentates is shown in (Table 2). Black tea bread (BT) had the highest antioxidant (3.1-fold, *p* = 0.0001) activity when compared with WB. Similarly, after digestion with α-amylase both breads containing BT showed higher antioxidant activity compared to βG (*p* = 0.0001). The antioxidant potential did not change significantly after gastric digestions for all breads. 

### 3.2. Short Chain Fatty Acid Production

#### 3.2.1. Fermentation pH 

There was no significant difference in pH between the breads at baseline, 6, and 24 h (Table 3). The pH was significantly (*p* = 0.001) lower at 6 h for all breads compared with 0 h in the range of 5.0 ± 0.8 to 5.5 ± 0.2. The pH remained significantly (*p* < 0.05) lower at 24 h after fermentation compared with baseline in the range of 5.2 ± 1.0 to 5.8 ± 0.4.

#### 3.2.2. Effects of Adding β-Glucan in Bread on SCFA Production

**Figure 2:**
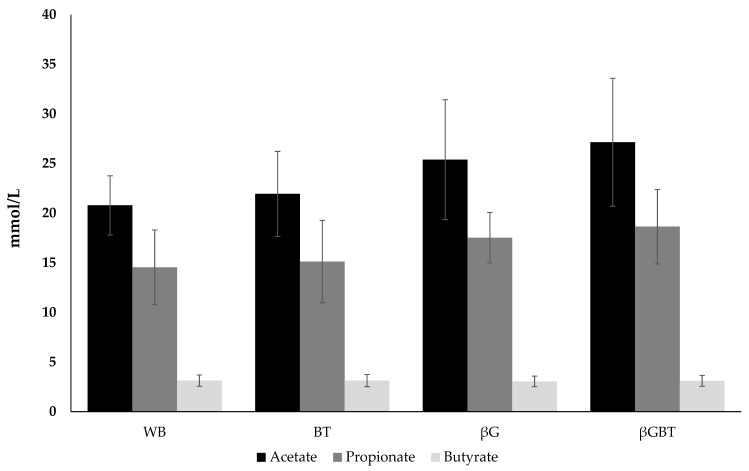
Production of SCFA (mmol/L) after 24 h fermentation of different breads after in vitro digestion. Results are mean (SD) (*n* = 4 donors). WB, white bread; BT, black tea bread; βG, β-glucan bread; βGBT, β-glucan with black tea bread.

SCFA production in breads containing β-glucan is shown in Figure 2. There was little impact of adding β-glucan in breads on individual SCFA compared with control WB. There were no significant differences in the production of acetate and propionate for BT, βG, and βGBT compared with control WB. The total ratio of individual SCFA (the sum of acetate, propionate and butyrate) of WB, BT, βG, and βGBT was comparable with 54:38:8, 55:37:8, 55:38:7, and 56:38:6, respectively.

#### 3.2.3. Donor SCFA Variability

**Figure 3:**
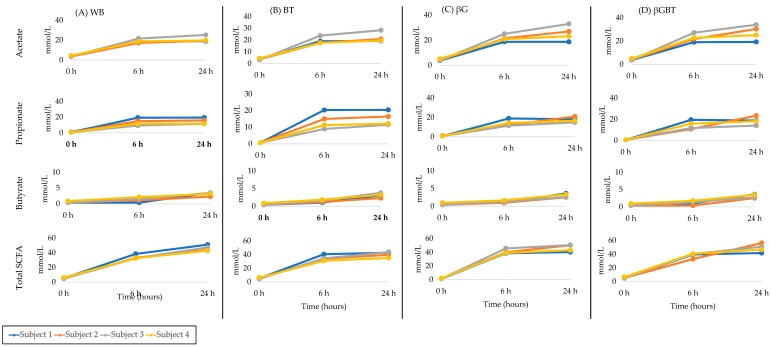
Absolute SCFA production in in vitro fermentations from individual Donors (*n* = 4) at 0, 6 and 24 h. (**A**) WB, white bread; (**B**) BT, black tea bread; (**C**) βG, beta glucan bread; (**D**) βGBT, beta glucan with black tea bread.

There was significant individual variability in SCFA production among donor samples (Figure 3). Donors 3 and 1 were consistently highest and lowest acetate producers, respectively, for all breads (Table 4). Donors 1 and 2 were consistently higher propionate producers for all breads compared with donor number 3. Donor number 1 was a higher butyrate producer compared with donor number 2 and 3.

## 4. Discussion

In this study, we investigated the effect of combining β-glucan and black tea in a food matrix (bread) to determine if there was a synergistic impact on total polyphenol and SCFA production by gut microbiota in an in vitro model of digestion and fermentation mimicking the human large intestine. In vitro digestion of BT bread with α-amylase increased polyphenol content and antioxidant potential compared with WB and βGBT breads. Tea polyphenols and high amylose maize starch form polyphenols-amylose complexes following thermal treatment [23]. The tea polyphenol-amylose complex modified the normal amylose retrogradation (re-crystalline) to a low-ordered crystalline structure. In another study, the addition of tea polyphenols (16%, *w*/*w* basis) prevented starch retrogradation [34]. Amylose and tea polyphenols are rich in hydroxyl groups, these hydrogen bonds govern the interaction between starch and tea polyphenols during gelatinization [34]. However, in our study, black tea was added to the bread mix. This will have different food matrix effects due to mixing, proofing, and baking. In previous studies, the addition of polyphenol extracts and pectin influenced the cross-linking of gluten leading to more water retention and changes in texture (softer bread) [35,36]. Therefore we hypothesized that adding black tea polyphenols to the bread mix will interact with gluten forming cross-links and a softer bread, which could expose starch granules to α-amylase activity and increased antioxidant potential of BT bread. On the other hand, in βGBT bread system, the interaction is much more complex than BT because of a food-matrix interaction between gluten, β-glucan, and black tea polyphenols. Simonsen et al. showed that tea polyphenols had the ability to form complexes with soluble β-glucan through hydrogen bonding [37]. This was further confirmed in an in vitro model system where the adsorption capacity of epigallocatechin gallate (EGCG), a major tea polyphenol, in the presence of β-glucan was clearly governed by hydrogen bonding [38,39]. According to this we expect that black tea polyphenols could form complexes with the gluten-network while β-glucan ‘preserves’ the starch structure for digestion by α-amylase. In our experiment, total polyphenols and antioxidant activity of βGBT remained higher compared with WB in an in vitro model mimicking small intestine enzymic digestion and absorption. The presence of polyphenols is of interest as they may be further metabolized by the colonic microbiota to phenolic acids [40]. However, a limitation of our study is the use of a simple in vitro total polyphenol method suitable for estimation of ‘crude’ polyphenols [41]. A more detailed analysis of phenolic acids using LC-MS would have provided a more comprehensive polyphenol profile.

In a human study where ileostomists ingested 452 μmol flavan-3-ols (from tea), it was shown that a large proportion (up to 73%) of flavan-3-ols pass from the small intestine to the colon [40]. Tea polyphenols are metabolized by colonic bacteria in the colon into SCFA and phenolic acids before being further metabolized in the liver or excreted [40]. The human gut microbiota metabolized tea polyphenols to 3-(3′-hydroxyphenyl)propionic and 3-(4′-hydroxyphenyl)propionic acids [42,43]. Liu et al. showed that gut microbiota degraded epigallocatechin gallate and epicatechin gallate and subsequently reduced their bioavailability [44]. However, the bioactivity of these phenolics acids in human health remains unknown and needs further investigation. Unno and Osakabe demonstrated that black tea supplementation in rats significantly reduced the relative abundance of *Clostridium* subcluster XIVa and *Clostridium* cluster XI compared with control [19]. Another study demonstrated that methylated (−)-epigallocatechin-3-*O*-(3-*O*-methyl) gallate (main component of tea polyphenols) reduced the *Firmicutes/Bacteroidetes* ratio in an experimental rat [45]. Still, it is still unknown whether the (polyphenol-linked β-glucan will be undigested and enter the large intestine to be metabolized by the bacteria to SCFA. To answer this question, the fermentability of the breads was investigated using an in vitro human colonic fermentation model.

This study showed that acetate and propionate were similar in the fermentation medium containing βG and βGBT compared to WB. Propionate production with βG was 17.5 mM and for βGBT 18.6 mM, with an acetate:propionate:butyrate production ratio of 56:38:6. This was similar to other in vitro studies of β-glucan either from oat or barley [6,46,47]. The propionate was in the range of 5.5–18 mM (acetate:propionate:butyrate ratio 51:32:17) considered propionate-rich. A previous study showed 2-fold individual differences in SCFA production [48]. In our study, donor number 3 consistently showed higher acetate and lower propionate production compared with donors number 1 and 2. This was similar to Harris et al., 2017 [49] who reported inter-individual variations in SCFA (acetate, propionate, and butyrate) with a range of 20 mmol/L to 100 mmol/L from 15 healthy Caucasians.

We have studied the fermentation of the predigested breads in an in vitro batch culture model, which is well established [30] but has several limitations common to all batch cultures. The products of fermentation are not removed and pH was not controlled, although the pH values reached in our study are representative of those found in the colon after ingestion of fermentable carbohydrates [31,32]. Continuous culture models, including a three-stage system representing proximal and distal colon environments [50] may be a better match to the human colon but these are expensive and impractical to use for multiple comparisons of food components. Indeed, most methods currently used have limitations in relation to host functionality [51].

An early study by Cummings et al. showed that dietary supplementation does not increase colonic production or circulating level of propionate due to variability in gut microbial activity [52]. Another study showed that an in vitro batch fermentation of inulin-propionate ester increased the microbial activity of *Bifidobacterium* spp, *Bacteroides*/*Prevotella* and *Atopobium* cluster compared with baseline (0 h) but did not differ significantly compared with the control (no substrate) and inulin-only control [53]. Inulin-propionate ester stimulated the release of appetite hormones (GLP-1 and PYY) and subsequently reduced energy intake among obese subjects [53]. However, the dose used was much higher than that produced in in vitro studies [46,47]. Propionate may activate G protein-coupled receptor 41, on colonic enteroendocrine L-cells [54,55] which are responsible for secretion of GLP-1 and PYY and may suppress food intake [54,55,56,57]. However, we failed to observe any synergistic effects of adding β-glucan in bread on SCFA production. This lack of effect could be partly explained by the context of the added β-glucan. β-glucan was not shown to increase product viscosity when added to solid food such as bread and biscuits unlike when added to liquid product [58]. Verbeke et al. using stable isotopes in vivo reported that less propionate was produced from barley kernel than with barley porridge (viscous liquid) [59]. This might be due to the lower water holding capacity of the barley kernel.

## 5. Conclusions

As expected, this study showed that BT bread had higher antioxidant activity and polyphenol content when digested with α-amylase compared with WB and βG. Antioxidant and polyphenol content of βGBT bread remained higher after in vitro small intestine digestion. β-glucan breads showed higher propionate concentrations at 24 h compared with baseline (0 h) but did not change significantly between breads. Black tea addition with β-glucan had no impact on SCFA production when added in bread. A higher dose of tea and β-glucan or combination with other soluble dietary fibers may be required to impact SCFA production.

## Figures and Tables

**Table 1:** Nutrient composition of experimental breads.

Ingredient (g)	White Bread (WB)	Black Tea Bread (BT)	β-Glucan Bread (βG)	β-Glucan + Black Tea Bread (βGBT)
**Strong white wheat flour**	500.0	500.0	500.0	500.0
**NaCl**	8.0	8.0	8.0	8.0
**Sugar**	6.0	6.0	6.0	6.0
**Dehydrated yeast**	8.0	8.0	10.0	10.0
**Butter (unsalted)**	6.0	6.0	6.0	6.0
**Skimmed milk powder**	7.0	7.0	7.0	7.0
**β-glucan**	0.0	0.0	35.0	35.0
**Black tea**	0.0	2.5	0.0	2.5
**Water (Ml)**	300	300	540	540
**Total**	835.0	837.5	1112.0	1114.5

**Table 2:** Total polyphenol (crude) content and antioxidant potential (FRAP assay) of retentates of breads prepared with black tea and β-glucan.

Bread	Total Polyphenols (μg GAE/g Retentate)		FRAP Activity of Retentate (μg Fe^2+^ Equivalents/g Retentate)	
	* Pre-Digested	** Digested	* Pre-Digested	** Digested
**WB**	222.6 ± 63.0 ^ade^	1077.7 ± 250.2 ^ab^	338.9 ± 131.2 ^ab^	3633.3 ± 645.0 ^a^
**BT**	361.1 ± 40.0 ^bc^	1228.9 ± 106.0 ^ac^	1056.2 ± 131.4 ^c^	4024.5 ± 198.8 ^a^
**βG**	229.1 ± 0.0 ^d^	1298.3 ± 276.0 ^ac^	236.2 ± 80.4 ^b^	3351.9 ± 552.2 ^a^
**βGBT**	291.5 ± 0.0 ^e^	1379.9 ± 146.8 ^c^	608.0 ± 119.0 ^d^	3998.8 ± 1030.4 ^a^

Asterisk (*) indicates predigested bread sample with α-amylase (0 h). Double asterisk (**) indicates digested bread sample with pepsin (2 h), pancreatin (5 h), and followed by dialysis for 6 h. Values with different letters (^a, b, c, d, e^) were significantly (*p* < 0.05) different between breads.

**Table 3:** Fermentation pH at 0, 6, and 24 h (mean + SD).

Bread	0 h	6 h	24 h
**WB**	6.6 ± 0.2	5.5 ± 0.2	5.8 ± 0.4
**BT**	6.6 ± 0.4	5.4 ± 0.4	5.7 ± 0.8
**βG**	6.6 ± 0.4	5.1 ± 0.8	5.3 ± 1.0
**βGBT**	6.6 ± 0.4	5.0 ± 0.8	5.2 ± 1.0

There were significant (*p* < 0.05) time interactions between 0 h vs. 6 h and 0 h vs. 24 h.

**Table 4:** Individual SCFA ranking by donor at end of fermentation of different breads after in vitro digestion (24 h).

Bread	Individual SCFA by donor			
	Acetate	Propionate	Butyrate	Total
**Donor 1**	BT = βGBT > WB > βG	BT > WB > βGBT > βG	βGBT = βG > WB > BT	BT > WB > βGBT > βG
**Donor 2**	βGBT > βG > BT > WB	βGBT > βG > BT > WB	βG > βGBT > BT > WB	βGBT > βG > BT > WB
**Donor 3**	βGBT > βG > BT > WB	βG > βGBT > BT > WB	BT > WB > βG > βGBT	βGBT > βG > BT > WB
**Donor 4**	βGBT > βG > WB > BT	βGBT > βG > BT > WB	βGBT > βG > BT > WB	βGBT > βG > BT > WB

WB, white bread; BT, black tea bread; βG, beta glucan bread; βGBT, beta glucan with black tea bread.

## References

[1] Rosell C.M., Preedy V.R., Watson R.R., Patel V.B. (2011). Chapter 1—The science of doughs and bread quality. Flour and Breads and Their Fortification in Health and Disease Prevention.

[2] Hayta M., Gamze Ö., Preedy V.R., Watson R.R., Patel V.B. (2011). Phytochemical fortification of flour and bread. Flour and Breads and Their Fortification in Health and Disease Prevention.

[3] Brennan C.S., Blake D.E., Ellis P.R., Schofield J.D. (1996). Effects of guar galactomannan on wheat bread microstructure and on the in vitro and in vivo digestibility of starch in bread. J. Cereal Sci..

[4] Jalil A.M., Edwards C.A., Combet E., Ibrahim M., Garcia A.L. (2015). Combined effects of added beta glucan and black tea in breads on starch functionality. Int. J. Food Sci. Nutr..

[5] Agostoni C., Bresson J.-L., Fairweather-Tait S., Flynn A., Golly I., Korhonen H., Lagiou P., Løvik M., Marchelli R., Martin A. (2011). Scientific opinion on the substantiation of health claims related to beta-glucans from oats and barley and maintenance of normal blood LDL-cholesterol concentrations (ID 1236, 1299), increase in satiety leading to a reduction in energy intake (ID 851, 852), reduction of post-prandial glycaemic responses (ID 821, 824), and “digestive function” (ID 850) pursuant to Article 13(1) of Regulation (EC) No 1924/20061. ESFA J..

[6] Panahi S., Ezatagha A., Jovanovski E., Jenkins A., Temelli F., Vasanthan T., Vuksan V. (2014). Glycemic effect of oat and barley beta-glucan when incorporated into a snack bar: A dose escalation study. J. Am. Coll. Nutr..

[7] Juvonen K.R., Salmenkallio-Marttila M., Lyly M., Liukkonen K.H., Lähteenmäki L., Laaksonen D.E., Uuusitupa M.I., Herzig K.H., Poutanen K.S., Karhunen L.J. (2011). Semisolid meal enriched in oat bran decreases plasma glucose and insulin levels, but does not change gastrointestinal peptide responses or short-term appetite in healthy subjects. Nutr. Metab. Cardiovasc. Dis..

[8] Hughes S.A., Shewry P.R., Gibson G.R., McCleary B.V., Rastall R.A. (2008). In vitro fermentation of oat and barley derived beta-glucans by human faecal microbiota. FEMS Microbiol. Ecol..

[9] Chambers E.S., Morrison D.J., Frost G. (2014). Control of appetite and energy intake by SCFA: What are the potential underlying mechanisms?. Proc. Nutr. Soc..

[10] Alhabeeb H., Chambers E.S., Frost G., Morrison D.J., Preston T. (2014). Inulin propionate ester increases satiety and decreases appetite but does not affect gastric emptying in healthy humans. Proc. Nutr. Soc..

[11] Li S.H., Zhao P., Tian H.B., Chen L.H., Cui L.Q. (2015). Effect of grape polyphenols on blood pressure: A Meta-analysis of randomized controlled trials. PLoS ONE.

[12] Chiva-Blanch G., Arranz S., Lamuela-Raventos R.M., Estruch R. (2013). Effects of wine, alcohol and polyphenols on cardiovascular disease risk factors: Evidences from human studies. Alcohol.

[13] Coe S., Ryan L. (2016). Impact of polyphenol-rich sources on acute postprandial glycaemia: A systematic review. J. Nutr. Sci..

[14] Pérez-Jiménez J., Díaz-Rubio M.E., Saura-Calixto F. (2013). Non-extractable polyphenols, a major dietary antioxidant: Occurrence, metabolic fate and health effects. Nutr. Res. Rev..

[15] Bamia C., Lagiou P., Jenab M., Trichopoulou A., Fedirko V., Aleksandrova K., Pischon T., Overvad K., Olsen A., Tjønneland A. (2016). Coffee, tea and decaffeinated coffee in relation to hepatocellular carcinoma in a European population: Multicentre, prospective cohort study. Int. J. Cancer.

[16] Deng Y.T., Lin-Shiau S.Y., Shyur L.F., Lin J.K. (2015). Pu-erh tea polysaccharides decrease blood sugar by inhibition of alpha-glucosidase activity in vitro and in mice. Food Funct..

[17] Satoh T., Igarashi M., Yamada S., Takahashi N., Watanabe K. (2015). Inhibitory effect of black tea and its combination with acarbose on small intestinal α-glucosidase activity. J. Ethnopharmacol..

[18] Hertog M.G.L., Hollman P.C.H., Putte B.V.D. (1993). Content of potentially anticarcinogenic flavonoids of tea infusions, wines, and fruit juices. J. Agric. Food Chem..

[19] Unno T., Osakabe N. (2018). Green tea extract and black tea extract differentially influence cecal levels of short-chain fatty acids in rats. Food Sci. Nutr..

[20] Stalmach A., Mullen W., Steiling H., Williamson G., Lean M.E., Crozier A. (2010). Absorption, metabolism, and excretion of green tea flavan-3-ols in humans with an ileostomy. Mol. Nutr. Food Res..

[21] Roowi S., Stalmach A., Mullen W., Lean M.E., Edwards C.A., Crozier A. (2010). Green tea flavan-3-ols: Colonic degradation and urinary excretion of catabolites by humans. J. Agric. Food Chem..

[22] Auger C., Mullen W., Hara Y., Crozier A. (2008). Bioavailability of polyphenon E flavan-3-ols in humans with an ileostomy. J. Nutr..

[23] Chai Y., Wang M., Zhang G. (2013). Interaction between amylose and tea polyphenols modulates the postprandial glycemic response to high-amylose maize starch. J. Agric. Food Chem..

[24] Rothwell J.A., Urpi-Sarda M., Boto-Ordoñez M., Knox C., Llorach R., Eisner R., Cruz J., Neveu V., Wishart D., Manach C. (2012). Phenol-Explorer 2.0: A major update of the Phenol-Explorer database integrating data on polyphenol metabolism and pharmacokinetics in humans and experimental animals. Database.

[25] Stewart A.J., Mullen W., Burns J., Lean M.E., Brighenti F., Crozier A. (2004). HPLC-MS analysis of phenolic compounds and purine alkaloids in green and black tea. J. Agric. Food Chem..

[26] Jacobs S.M., Izydorczyk M.S., Preston K.R., Dexter J.E. (2008). Evaluation of baking procedures for incorporation of barley roller milling fractions containing high levels of dietary fibre into bread. J. Sci. Food Agric..

[27] Aura A.-M., Härkönen H., Fabritius M., Poutanena K. (1999). Development of an in vitro enzymic digestion method for removal of starch and protein and assessment of its performance using rye and wheat breads. J. Cereal Sci..

[28] Slinkard K., Singleton V.L. (1977). Total phenol analyses: Automation and comparison with manual methods. Am. J. Enol. Vitic..

[29] Benzie I.F., Strain J.J. (1996). The ferric reducing ability of plasma (FRAP) as a measure of “antioxidant power”: The FRAP assay. Anal. Biochem..

[30] Jaganath I., Mullen W., Lean M., Edwards C., Crozier A. (2009). In vitro catabolism of rutin by human fecal bacteria and the antioxidant capacity of its catabolites. Free Radic. Biol. Med..

[31] Metzler-Zebeli B.U., Canibe N., Montagne L., Freire J. (2019). Resistant starch reduces large intestinal pH and promotes fecal lactobacilli and bifidobacterial in pigs. Animal.

[32] Bown R.L., Gibson J.A., Sladen G.E., Hicks B., Dawson A.M. (1974). Effects of lactulose and other laxatives on ileal and colonic pH as measured by a radiotelemetry device. Gut.

[33] Laurentin A., Edwards C.A. (2004). Differential fermentation of glucose-based carbohydrates in vitro by human faecal bacteria--a study of pyrodextrinised starches from different sources. Eur. J. Nutr..

[34] Wu Y., Lin Q., Chen Z., Xiao H. (2011). The interaction between tea polyphenols and rice starch during gelatinization. Food Sci. Technol. Int..

[35] Sivam A.S., Sun-Waterhouse D., Waterhouse G.I., Quek S., Perera C.O. (2011). Physicochemical properties of bread dough and finished bread with added pectin fiber and phenolic antioxidants. J. Food Sci..

[36] Sun-Waterhouse D., Chen J., Chuah C., Wibisono R., Melton L.D., Laing W., Ferguson L.R., Skinner M.A. (2009). Kiwi fruit-based polyphenols and related antioxidants for functional foods: Kiwi fruit extract-enhanced gluten-free bread. Int. J. Food Sci. Nutr..

[37] Simonsen H.T., Nielsen M.S., Christensen N.J., Christensen U., La Cour T.V., Motawia M.S., Jespersen B.P., Engelsen S.B., Møller B.L. (2009). Molecular interactions between barley and oat beta-glucans and phenolic derivatives. J. Agric. Food Chem..

[38] Wang Y., Liu J., Chen F., Zhao G. (2013). Effects of molecular structure of polyphenols on their noncovalent interactions with oat beta-glucan. J. Agric. Food Chem..

[39] Wu Z., Ming J., Gao R., Wang Y., Liang Q., Yu H., Zhao G. (2011). Characterization and antioxidant activity of the complex of tea polyphenols and oat beta-glucan. J. Agric. Food Chem..

[40] Stalmach A., Troufflard S., Serafini M., Crozier A. (2009). Absorption, metabolism and excretion of Choladi green tea flavan-3-ols by humans. Mol. Nutr. Food Res..

[41] Granatoa D., Shahidi F., Wrolstad R., Kilmartin P., Melton L.D.T., Hidalgo F.J., Miyashita K., van Camp J., Alasalvar C., Ismail A. (2018). Antioxidant activity, total phenolics and flavonoids contents: Should we ban in vitro screening methods?. Food Chem..

[42] Parkar S.G., Trower T.M., Stevenson D.E. (2013). Fecal microbial metabolism of polyphenols and its effects on human gut microbiota. Anaerobe.

[43] Goodwin B.L., Ruthven C.R., Sandler M. (1994). Gut flora and the origin of some urinary aromatic phenolic compounds. Biochem. Pharmacol..

[44] Liu A.B., Tao S., Lee M.-J., Hu Q., Meng X., Lin Y., Yang C.S. (2018). Effects of gut microbiota and time of treatment on tissue levels of green tea polyphenols in mice. Biofactors.

[45] Zhang X., Chen Y., Zhu J., Zhang M., Ho C.-T., Huang Q., Cao J. (2018). Metagenomics analysis of gut microbiota in a high fat diet-induced obesity mouse model fed with (-)-epigallocatechin 3-*O*-(3-*O*-methyl) gallate (EGCG3’’Me). Mol. Nutr. Food Res..

[46] Nordlund E., Aura A.M., Mattila I., Kössö T., Rouau X., Poutanen K. (2012). Formation of phenolic microbial metabolites and short-chain fatty acids from rye, wheat, and oat bran and their fractions in the metabolical in vitro colon model. J. Agric. Food Chem..

[47] Kim H.J., White P.J. (2011). Optimizing the molecular weight of oat beta-glucan for in vitro bile acid binding and fermentation. J. Agric. Food Chem..

[48] Carlson J., Esparza J., Swan J., Taussig D., Combs J., Slavin J. (2016). In vitro analysis of partially hydrolyzed guar gum fermentation differences between six individuals. Food Funct..

[49] Harris H.C., Edwards C.A., Morrison D.J. (2017). Impact of glycosidic bond configuration on short chain fatty acid production from model fermentable carbohydrates by the human gut microbiota. Nutrients.

[50] Macfarlane G.T., Macfarlane S., Gibson G.R. (1998). Validation of a three-stage compound continuous culture system for investigating the effect of retention time on the ecology and metabolism of bacteria in the human colon. Microb. Ecol..

[51] Moon J.S., Li L., Bang J., Han N.S. (2016). Application of in vitro gut fermentation models to food components: A review. Food Sci. Biotechnol..

[52] Cummings J.H., Pomare E.W., Branch W.J., Naylor C.P.E., Macfarlane G.T. (1987). Short chain fatty acids in human large intestine, portal, hepatic and venous blood. Gut.

[53] Chambers E.S., Viardot A., Psichas A., Morrison D.J., Murphy K.G., Zac-Varghese S.E., MacDougall K., Preston T., Tedford C., Finlayson G.S. (2015). Effects of targeted delivery of propionate to the human colon on appetite regulation, body weight maintenance and adiposity in overweight adults. Gut.

[54] Psichas A., Sleeth M.L., Murphy K.G., Brooks L., Bewick G.A., Hanyaloglu A.C., Ghatei M.A., Bloom S.R., Frost G. (2015). The short chain fatty acid propionate stimulates GLP-1 and PYY secretion via free fatty acid receptor 2 in rodents. Int. J. Obes. (Lond.).

[55] Karaki S., Tazoe H., Hayashi H., Kashiwabara H., Tooyama K., Suzuki Y., Kuwahara A. (2008). Expression of the short-chain fatty acid receptor, GPR43, in the human colon. J. Mol. Histol..

[56] Murphy K.G., Dhillo W.S., Bloom S.R. (2006). Gut peptides in the regulation of food intake and energy homeostasis. Endocr. Rev..

[57] Batterham R.L., Cowley M.A., Small C.J., Herzog H., Cohen M.A., Dakin C.L., Wren A.M., Brynes A.E., Low M.J., Ghatei M.A. (2002). Gut hormone PYY (3-36) physiologically inhibits food intake. Nature.

[58] Åman P., Lena R., Roger A. (2004). Molecular weight distribution of beta-glucan in oat-based foods. Cereal Chem..

[59] Verbeke K., Ferchaud-Roucher V., Preston T., Small A.C., Henckaerts L., Krempf M., Wang H., Vonk R.J., Priebe M.G. (2010). Influence of the type of indigestible carbohydrate on plasma and urine short-chain fatty acid profiles in healthy human volunteers. Eur. J. Clin. Nutr..

